# Engineering Limonene and Bisabolene Production in Wild Type and a Glycogen-Deficient Mutant of *Synechococcus* sp. PCC 7002

**DOI:** 10.3389/fbioe.2014.00021

**Published:** 2014-06-19

**Authors:** Fiona K. Davies, Victoria H. Work, Alexander S. Beliaev, Matthew C. Posewitz

**Affiliations:** ^1^Department of Chemistry and Geochemistry, Colorado School of Mines, Golden, CO, USA; ^2^Civil and Environmental Engineering Division, Colorado School of Mines, Golden, CO, USA; ^3^Biological Sciences Division, Pacific Northwest National Laboratory, Richland, WA, USA

**Keywords:** terpenoids, limonene, bisabolene, cyanobacteria, glycogen, *glgC*, metabolic sink

## Abstract

The plant terpenoids limonene (C_10_H_16_) and α-bisabolene (C_15_H_24_) are hydrocarbon precursors to a range of industrially relevant chemicals. High-titer microbial synthesis of limonene and α-bisabolene could pave the way for advances in *in vivo* engineering of tailor-made hydrocarbons, and production at commercial scale. We have engineered the fast-growing unicellular euryhaline cyanobacterium *Synechococcus* sp. PCC 7002 to produce yields of 4 mg L^−1^ limonene and 0.6 mg L^−1^ α-bisabolene through heterologous expression of the *Mentha spicata* l-limonene synthase or the *Abies grandis* (E)-α-bisabolene synthase genes, respectively. Titers were significantly higher when a dodecane overlay was applied during culturing, suggesting either that dodecane traps large quantities of volatile limonene or α-bisabolene that would otherwise be lost to evaporation, and/or that continuous product removal in dodecane alleviates product feedback inhibition to promote higher rates of synthesis. We also investigate limonene and bisabolene production in the Δ*glgC* genetic background, where carbon partitioning is redirected at the expense of glycogen biosynthesis. The *Synechococcus* sp. PCC 7002 Δ*glgC* mutant excreted a suite of overflow metabolites (α-ketoisocaproate, pyruvate, α-ketoglutarate, succinate, and acetate) during nitrogen-deprivation, and also at the onset of stationary growth in nutrient-replete media. None of the excreted metabolites, however, appeared to be effectively utilized for terpenoid metabolism. Interestingly, we observed a 1.6- to 2.5-fold increase in the extracellular concentration of most excreted organic acids when the Δ*glgC* mutant was conferred with the ability to produce limonene. Overall, *Synechococcus* sp. PCC 7002 provides a highly promising platform for terpenoid biosynthetic and metabolic engineering efforts.

## Introduction

In recent years, cyanobacteria have been successfully used as a platform to generate a range of commercially significant products, including isoprene (Lindberg et al., 2010; Bentley and Melis, 2012; Bentley et al., 2014), isobutanol (Atsumi et al., 2009), 2,3-butanediol (Oliver et al., 2013, 2014), and ethylene (Ungerer et al., 2012). In all cases, photoassimilated carbon was drawn from native metabolic pathways through the heterologous expression of one or more enzymes to create new carbon sinks. While demonstrating the proof-of-principle, these strategies are unlikely to attain high yields of novel end-products as carbon partitioning and regulation are hardwired into the organism’s central metabolic networks. For cyanobacteria, one of the main metabolic engineering bottlenecks is the ability to redirect carbon from the major glycogen and protein sinks to pathways of interest. A careful rewiring of central metabolism is required to increase product yield at the expense of biomass accumulation, without negatively influencing photosynthesis and carbon fixation rates.

**Figure 1:**
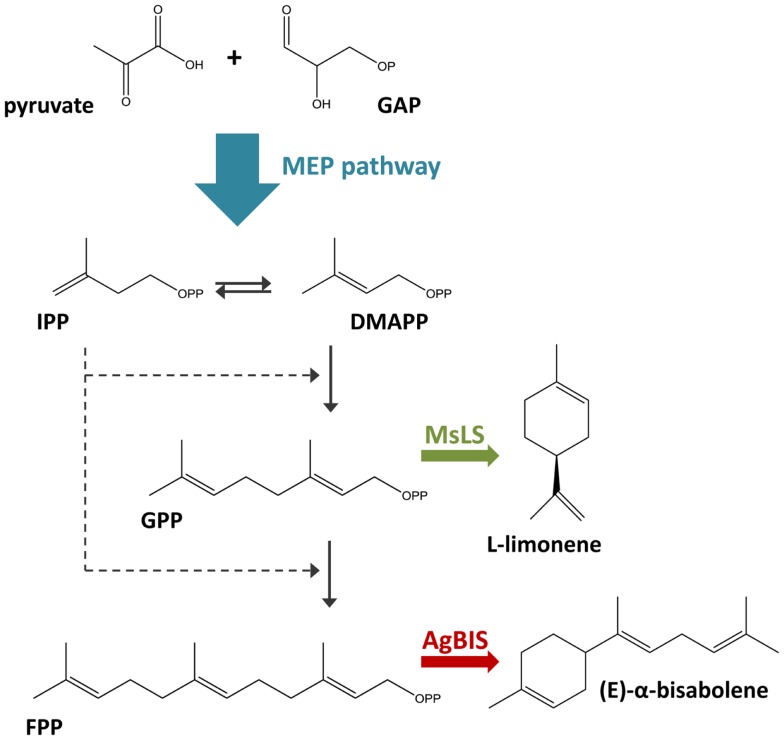
**Terpenoid biosynthesis via the 2-C-methyl-l-erythritol 4-phosphate (MEP) pathway**. The MEP terpenoid biosynthetic pathway converts pyruvate and glyceraldehyde-3-phosphate (GAP) feedstock to the C5 isomers, isopentenyl pyrophosphate (IPP), and dimethylallyl pyrophosphate (DMAPP). The stepwise addition of IPP to DMAPP generates C10 geranyl pyrophosphate (GPP) and C15 farnesyl pyrophosphate (FPP), the respective precursors to monoterpenes and sesquiterpenes. In this study, the heterologous expression of the *M. spicata* l-limonene synthase (MsLS) and *A. grandis* (E)-α-bisabolene synthase (AgBIS) in *Synechococcus* sp. PCC 7002 was designed to draw on GPP and FPP pools, respectively, generated by the native cyanobacterial MEP pathway for the production of the non-native terpenoids l-limonene and α-bisabolene.

In the context of photoautotrophic biotechnology, the terpenoid biosynthetic pathway is of particular interest, as it produces the largest and most diverse array of naturally occurring organic compounds (typically of plant origin) (Davies et al., 2014). The plant terpenoids limonene (C_10_H_16_) and bisabolene (C_15_H_24_) are recognized as precursors to a range of commercially valuable products, with applications in biofuels, bioplastics, pharmaceutical, nutraceutical, and cosmetic industries (Duetz et al., 2003; Peralta-Yahya et al., 2011). In this study, we engineer the fast-growing euryhaline cyanobacterium *Synechococcus* sp. PCC 7002 for the production of limonene and α-bisabolene through heterologous expression of the *Mentha spicata* l-limonene synthase and *Abies grandis* (E)-α-bisabolene synthase genes. In cyanobacteria, terpenoids are synthesized via the 2-*C*-methyl-l-erythritol 4-phosphate **(**MEP) pathway, utilizing photosynthetically generated pyruvate and glyceraldehyde-3-phosphate (GAP) as the primary substrates (Figure 1). Limonene and bisabolene represent new carbon sinks that draw from the native terpenoid pathway at the levels of geranyl pyrophosphate (GPP) and farnesyl pyrophosphate (FPP).

The two major competing pathways for cyanobacterial terpenoid production are protein and glycogen (the cyanobacterial storage carbohydrate) biosynthesis. Recent studies in *Synechocystis* sp. PCC 6803 and *Synechococcus* sp. PCC 7942 have demonstrated that upon simultaneous inactivation of protein and glycogen sinks (*via* nitrogen-deprivation in the Δ*glgC* glycogen-deficient mutant), a suite of organic acids are excreted due to the resulting metabolic imbalance (Carrieri et al., 2012; Grundel et al., 2012; Hickman et al., 2013). In our study, we sought to elucidate how such major shifts in flux distribution will affect the overall metabolic partitioning toward the terpenoid pathway in *Synechococcus* sp. PCC 7002, using the newly engineered limonene and α-bisabolene terpenoid sinks as the reporting products.

## Materials and Methods

### Cloning and plasmid construction

The *E. coli* DH5α strain was used for routine subcloning according to standard protocols. The (−)-4*S*-limonene synthase gene from *M. spicata* (GenBank accession Q40322) (Colby et al., 1993) was optimized for codon-usage in *Synechococcus* sp. PCC 7002 (DNA 2.0, USA) without the predicated chloroplast transit peptide (cTP), and is referred to in this work as MsLS. The first 168 bases of Q40322 encoding the cTP were replaced with an ATG codon for translation initiation in *Synechococcus* sp. PCC 7002, retaining residues R58/R59 that are required for catalytic activity (Williams et al., 1998). The (*E*)-α-bisabolene synthase gene from *A. grandis* (GenBank accession O81086) (Bohlmann et al., 1998; Trapp and Croteau, 2001) was also optimized for codon-usage in *Synechococcus* sp. PCC 7002 (DNA 2.0, USA), and is referred to as AgBIS. Oligonucleotide primers used to amplify these codon-optimized genes are as follows (introduced restriction sites for cloning are indicated in bold, and lower case letters represent start or stop codons): MsLS, MsLS_**NcoI**_F, 5′-CTTCGCTGAA**CCatgG**AACGTCGCTC-3′, and MsLS_**BamHI**_R, 5′-TGACG**GGATCC**ttaAGCGAAGG-3′; AgBIS, AgBIS_**NcoI**_F, 5′-AGATTAATT**CCatgG**CCGGTGTGA-3′; and AgBIS_**BamHI**_R, 5′-GCC**GGATCC**ttaTAACGGCAAC-3′.

A neutral site (NSI) for the integration of transgenes was identified in the *Synechococcus* sp. PCC 7002 genome between two open-reading frames encoding hypothetical proteins (SYNPCC7002_A0935 and SYNPCC7002_A0936), which we have termed NSI. This was used as the site of integration for the limonene synthase (MsLS) and α-bisabolene synthase (AgBIS) genes. Oligonucleotide primers designed to amplify 750 bp up-stream (us) and down-stream (ds) regions flanking NSI, which were used for double homologous recombination in *Synechococcus* sp. PCC 7002 are as follows (introduced restriction sites are indicated in bold): NSIus, 935us_**NsiI**_F, 5′-AGTTCAC**ATGCAT**AAAGTCAA-3′, and 935us_**EcoRI**_R, 5′-CGTATAG**GAATTC**TTACTCAG-3′; NSIds, 936ds_**SalI**_F, 5′-GCATACT**GTCGAC**CTATTTTAT-3′; and 936ds_**SphI**_R, 5′-AGTTGAC**GCATGC**AGAGGTGG-3′. These regions were cloned into a pAQ1 plasmid containing the *Synechocystis* sp. PCC 6803 *cpcBA* (*cpc*) promoter (Xu et al., 2011). The MsLS and AgBIS transgenes, along with a spectinomycin-resistance cassette (SmR), were cloned between the NSI-flanking regions immediately down-stream of the *cpc* promoter, generating plasmids pNSI-cpc-MsLS-SmR and pNSI-cpc-AgBIS-SmR. To demonstrate that any phenotypes arising from the expression of MsLS or AgBIS were not a direct result of interference with the NSI region of *Synechococcus* sp. PCC 7002 genomic DNA, we also constructed the control plasmid pNSI-cpc-YFP-SmR, containing a YFP gene in place of the MsLIM or AgBIS terpene synthase (TPS) genes.

### Cyanobacterial strains, growth conditions, and transformation

**Figure 2:**
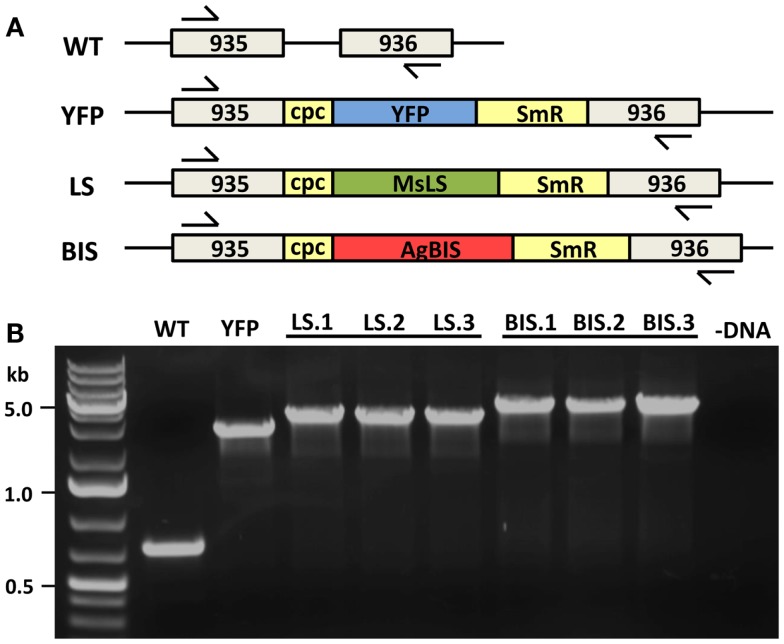
**MsLS and AgBIS transgene integration to the *Synechococcus* chromosome**. **(A)** The YFP, MsLS, and AgBIS transgene constructs were introduced to a neutral site (NSI) of genomic DNA (between open-reading frames A0935 and A0936) via double homologous recombination. Constructs contained the *Synechocystis* sp. PCC 6803 *cpcBA* (*cpc*) promoter to drive transgene expression, and a spectinomycin-resistance selectable marker (SmR). Arrows indicate the position of genomic DNA primers used to check for integration and complete chromosomal segregation in transformant lines. **(B)** Verification of compete chromosomal segregation for the introduced transgenes in YFP, LS, and BIS transformants by colony-PCR using the above-mentioned primers. Three independent transformant lines of LS (LS.1, LS.2, and LS.3) and BIS (BIS.1, BIS.2, and BIS.3) were verified.

Wild type and transformant *Synechococcus* sp. PCC 7002 strains were maintained on solid A^+^ media (Stevens et al., 1973) supplemented with 8.25 mM Tris–HCl (pH 8.2) and 0.3% sodium thiosulfate. Where appropriate, spectinomycin was used at a concentration of 50 μg mL^−1^ and kanamycin at 100 μg mL^−1^. Liquid cultures were grown in A^+^ media supplemented with 8.25 mM Tris–HCl (pH 8.2). For nitrogen-deprivation, NaNO_3_ was omitted from the growth media and replaced mol:mol with NaCl. Nitrogen-deplete media is referred to as A^+^(−N). Cultures were grown in 250 mL Erlenmeyer flasks on an orbital shaker at 37°C, in an incubator with an atmosphere of 1% CO_2_ in air, and constant illumination at 250 μmol photons m^−2^ s^−1^ of photosynthetically active radiation (PAR). Liquid starter cultures were grown in the presence of antibiotics; however, during experimental procedures antibiotics were eliminated. Transformation of *Synechococcus* sp. PCC 7002 with the plasmid constructs described above was performed according to established procedures for cyanobacteria (Eaton-Rye, 2011) to generate the strains LS (containing MsLS) and BIS (containing AgBIS). Complete chromosomal segregation for the introduced transgenes was achieved through propagation of multiple generations on selective agar and verified by colony-PCR using the primers: NSI_us_F, 5′-CTAGCACAAATGAAGCCCGAC-3′, and NSI_ds_R, 5′-GCAGATATAAGCAACGGTACAG-3′ (Figure 2). The Δ*glgC* strain was obtained from D. A. Bryant, which was generated via the insertional disruption of the *glgC* open reading frame (SYNPCC7002_A0095) with a kanamycin-resistance cassette (KmR) (Guerra et al., 2013). Oligonucleotide primers used to verify *glgC* disruption in transformant strains are: glgC_F, 5′-TCACGTAGTCGGGTTTGATGTC-3′, and glgC_R, 5′-CACTAAAGTCCACGACACGACC-3′. The Δ*glgC* strain was also transformed with the MsLS and AgBIS plasmid constructs to generate strains Δ*glgC*:LS and Δ*glgC*:BIS, respectively.

### Terpenoid extraction and quantification

*Synechococcus* cultures used for terpenoid production assays were grown photoautotrophically in 100 mL batches, with an inoculation density of OD_730 nm_ = 2.0. Where indicated, a 5-mL dodecane (analytical grade, Sigma-Aldrich, USA) overlay was applied to the culture at *t* = 0 to trap terpenoids during the 96-h experimental time course. At 96 h, cells were harvested by centrifugation to separate the biomass from the media. Terpenoid extraction was performed on the cell pellet using the chloroform:methanol:water biphasic separation protocol described by Bligh and Dyer (1959). To determine if any accumulated terpenoids had partitioned to the media or adhered to the flask walls, the supernatant was returned to the original Erlenmeyer flask and a 5-mL dodecane overlay applied for 1 h for terpenoid extraction.

Aliquots of 200 μL were harvested from the dodecane overlay at each experimental time point. These samples, and the chloroform extracts from the cell pellet, were analyzed directly using 1 μL of sample injected into a GC-FID (Agilent 7890A) equipped with a DB-5-ms column (Agilent Technologies, Santa Clara, CA, USA; 30 m × 0.25 mm; 0.25 μm film thickness) and operated using the temperature program: 60–310°C (at 12°C min^−1^), and H_2_ carrier gas at 1.5 mL min^−1^. Quantification of limonene or α-bisabolene from the dodecane or chloroform extracts was performed using standard curves constructed by GC peak integration of serial dilutions of limonene (Sigma-Aldrich, USA) and bisabolene (Alfa Aesar, USA) standards in the appropriate solvent. Due to the reverse solvent effect (Grob, 1983; Grob and Schilling, 1983), limonene was observed as a broad peak on the chromatogram when mixed with dodecane. Dilution of the sample with a solvent of lower molecular weight than the solute typically eliminates this problem. However, dilution pushed limonene below the limits of detection in some samples, and so limonene peaks were integrated using the area measured under the broad limonene peak. The bisabolene standard contained a number of discrete peaks, and ~40% of the total combined peak areas were attributed to other sesquiterpenes with different retention times relative to α-bisabolene. The weight contribution by α-bisabolene was adjusted accordingly for the standard curves. Mass spectral analysis was conducted using a Varian 3800 GC and Varian 1200 quadrupole MS/MS equipped with a Rxi-5ms column (30 m × 0.25 mm; 0.25 μm film thickness); temperature program: 60–310°C (at 12°C min^−1^); He carrier gas at 1.2 mL min^−1^; mass spectra, 70 eV, EI mode; ion source temperature 215°C; scan mass range, 18–500.

### Excreted organic acid measurements

*Synechococcus* cultures used for excreted metabolite assays were grown photoautotrophically in 50 mL batches with an inoculation density of OD_730 nm_ = 2.0. Cells were washed twice with A^+^(−N) media to remove media nitrogen before inoculation in either nitrogen-replete or nitrogen-deplete media for experimentation. Aliquots of cell suspension (1 mL) were removed from the culture at appropriate time points and centrifuged to pellet cell biomass. The resulting supernatant was filtered through a 0.45-μm silicon membrane and the filtrate used directly for high-performance liquid chromatography (HPLC) and nuclear magnetic resonance (NMR) spectroscopy. A Surveryor Plus (Thermo Scientific, Waltham MA) HPLC equipped with an Aminex fermentation monitoring column [(150 mm × 7.8 mm); Bio-Rad, Hercules, CA, USA] was used for HPLC analyses. 25 μL of filtrate was injected for isocratic elution using 8 mM H_2_SO_4_ as the mobile phase with a flow rate of 0.5 mL min^−1^ and a column operating temperature of 45°C. A refractive index (RI) detector operating at 50°C was used for metabolite identification and quantification. Metabolite peaks were integrated and quantified using standard curves constructed from serial dilutions of authentic standards (Sigma-Aldrich, USA). Proton NMR spectra were generated with a JEOL ECA 500 MHz spectrometer; 128 scans were used with water-suppression.

### Biomass, chlorophyll *a*, carbohydrate, and whole cell absorption spectra measurements

Cyanobacterial biomass accumulation was measured as dry cell weight (DCW), where 2 mL samples of culture were pelleted and washed with 8.25 mM Tris–HCl (pH 8.2) to remove any residual salt from the growth medium. Pellets were then resuspended in 2 mL 8.25 mM Tris (pH 8.2), and dried overnight at 80°C in pre-weighed aluminum dishes prior to measuring the DCW. The dry weight of 2 mL 8.25 mM Tris (pH 8.2) solution (without cells) was subtracted from the cell dry weight to eliminate Tris buffer contributions. Chlorophyll *a* concentrations in cell suspensions were determined spectrophotometrically in 90% methanol extracts according to Meeks and Castenholz (1971). Total reducing sugars (in glucose equivalents) were quantified by the anthrone assay, as described by Meuser et al. (2011), as a measure of the carbohydrate content of cells. For whole cell absorption spectra, cell suspensions were normalized to an OD_730 nm_ of 0.3 and measured spectrophotometrically (400–800 nm).

## Results

### Construction of *Synechococcus* sp. PCC 7002 strains carrying MsLS and AgBIS transgenes

A “NSI” region of the *Synechococcus* sp. PCC 7002 genome was identified between two open reading frames encoding hypothetical proteins and used as a site for transgene integration (described in Materials and Methods). The *cpcBA* promoter from *Synechocystis* sp. PCC 6803 (Xu et al., 2011) was used to drive expression of the TPS transgenes. Numerous lines were selected for each new strain generated to ensure that any phenotypes arising were a consistent feature of the specific transformation. Figure 2 shows the successful integration of the MsLS and AgBIS constructs at the NSI locus, as evidenced by the gel shift of the amplified colony-PCR product in transformant strains relative to the wild type. Integration of the YFP construct was also confirmed in the YFP transformant, which was designed as a control strain to identify phenotypes in the LS and BIS strains that may be attributed to chromosomal disruption at the NSI locus, rather than expression of the TPS transgenes. Importantly, the lack of a wild type-sized band in all transformant lines indicated that homoplasmy for the introduced transgenes was achieved.

### Identification of limonene and α-bisabolene as end-products of transformant strains

**Figure 3:**
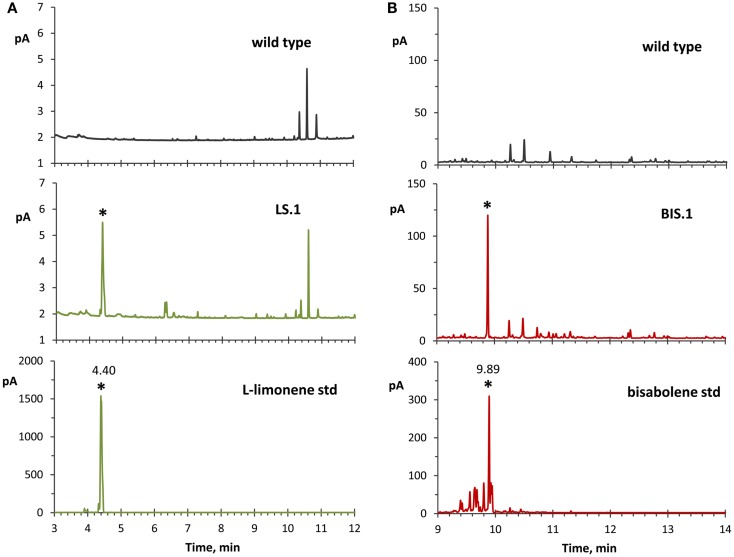
**GC–FID analyses of solvent overlay-extracted limonene and bisabolene from transformant strains**. **(A)** Hexane-overlay extraction from the LS transformant (LS.1 line as a representative) showed accumulation of a product that was absent in the wild type, and with a similar retention time (4.40 min) to a commercial l-limonene standard. **(B)** Dodecane overlay extraction from the BIS transformant (BIS.1 line as a representative) revealed a peak with a similar retention time (9.89 min) to a bisabolene standard, which is absent in the wild type. For these experiments, dodecane was used for bisabolene extraction because of the extended period of time required to accumulate detectable levels of bisabolene, whereas hexane was applied for better limonene peak resolution due to the reverse solvent effect observed with dodecane.

**Figure 4:**
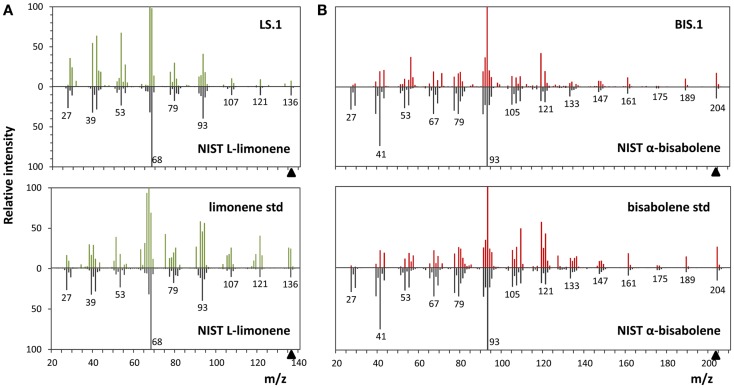
**Mass spectra analyses of limonene and α-bisabolene**. **(A)** Comparison of the l-limonene reference spectra from the NIST library with the spectra of the putative l-limonene peak in the LS.1 line (upper panel) and the authentic l-limonene standard (lower panel). **(B)** α-Bisabolene NIST reference spectra alignment with the putative α-bisabolene peak in the BIS.1 line (upper panel) and a commercial bisabolene standard (lower panel).

The use of an organic solvent overlay has proven to be a successful method for harvesting terpenoids from microbial cultures (Newman et al., 2006; Anthony et al., 2009; Alonso-Gutierrez et al., 2013; Bentley et al., 2013). GC–FID analyses of solvent overlays from LS (Figure 3A) and BIS (Figure 3B) transformants showed prominent peaks with similar retention times to commercial standards of l-limonene (4.40 min) and bisabolene (9.89 min), respectively, which were absent from the wild type. Figure 4 shows the mass spectral analyses of putative l-limonene and α-bisabolene peaks from transformant solvent extracts in comparison to the corresponding reference spectra from the NIST Mass Spectral Library (upper panels). The solvent also extracted additional products common to all strains, including wild type (Figure 3, ~10.5 min retention time), which may be membrane lipids (accumulated levels were below detection limits for mass spectral analysis). A comparison of l-limonene and bisabolene commercially available standards with NIST reference spectra is also shown in Figure 4 (lower panels). The major peak (9.89 min) in the bisabolene standard (Figure 3B) was identified as α-bisabolene, the peak at 9.94 min was bisabolol, while the small peaks with retention times <9.98 min were all sesquiterpenes based on a mass to charge (*m*/*z*) of 204.

### Transformant fitness and effect of dodecane on the rates of photoautotrophic growth

**Figure 5:**
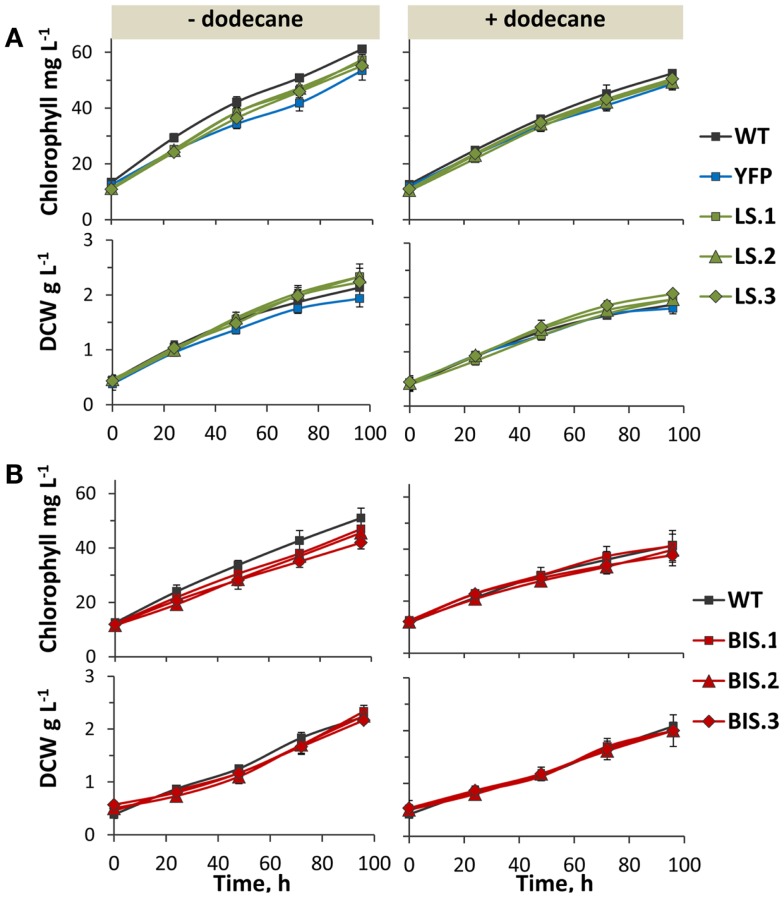
**Photoautotrophic growth of transformant lines in the presence and absence of a dodecane overlay**. **(A)** Chlorophyll and biomass accumulation [as measured by dry cell weight (DCW)] over 96 h in the YFP control strain, and three independent LS lines (LS.1, LS.2, and LS.3) in comparison to wild type. Left panels show growth in the absence of a solvent overlay, while right panels display growth in the presence of a dodecane overlay. **(B)** Comparison of chlorophyll and biomass accumulation (DCW) between wild type and three independent BIS transformant lines (BIS.1, BIS.2, and BIS.3), in the absence (left panels) and presence (right panels) of a dodecane overlay. Error bars represent standard deviation from at least three biological replicates.

Photoautotrophic growth rates, as measured by chlorophyll and biomass accumulation, were comparable between wild type and the YFP control strain (Figure 5A, left panels). This verified that NSI is a suitable “neutral” site for transgene integration in *Synechococcus* sp. PCC 7002 under the defined experimental conditions, because cell growth rate was not adversely affected by the genetic manipulation at this locus. Rates of chlorophyll and biomass accumulation in the LS and BIS transformants were also comparable to that of the wild type (Figure 5, left panels), confirming that expression of the MsLS and AgBIS transgenes, and subsequent intracellular accumulation of limonene and α-bisabolene, did not adversely affect cell fitness under our experimental conditions.

Dodecane was chosen as the solvent of choice for longer-term terpenoid extraction experiments due to its relatively low volatility, which enabled continuous extraction over multiple days (evaporative loss was determined as negligible over the five day cultivation period, data not shown). The C_12_ chain length also allowed chromatographic separation from C_10_ limonene and C_15_ bisabolene. The rate of limonene and α-bisabolene biosynthesis during the growth phase was measured over a 96-h time course using a 5% (v/v) dodecane overlay. Notably, the presence of the dodecane overlay did not greatly affect chlorophyll content or biomass yield in any of the strains (Figure 5, right panels), indicating that atmospheric CO_2_ was able to efficiently diffuse though the dodecane layer and dissolve in the liquid media at concentrations that were not limiting for photoautotrophic growth under these conditions.

### Limonene and bisabolene biosynthetic rates and cellular localization

**Figure 6:**
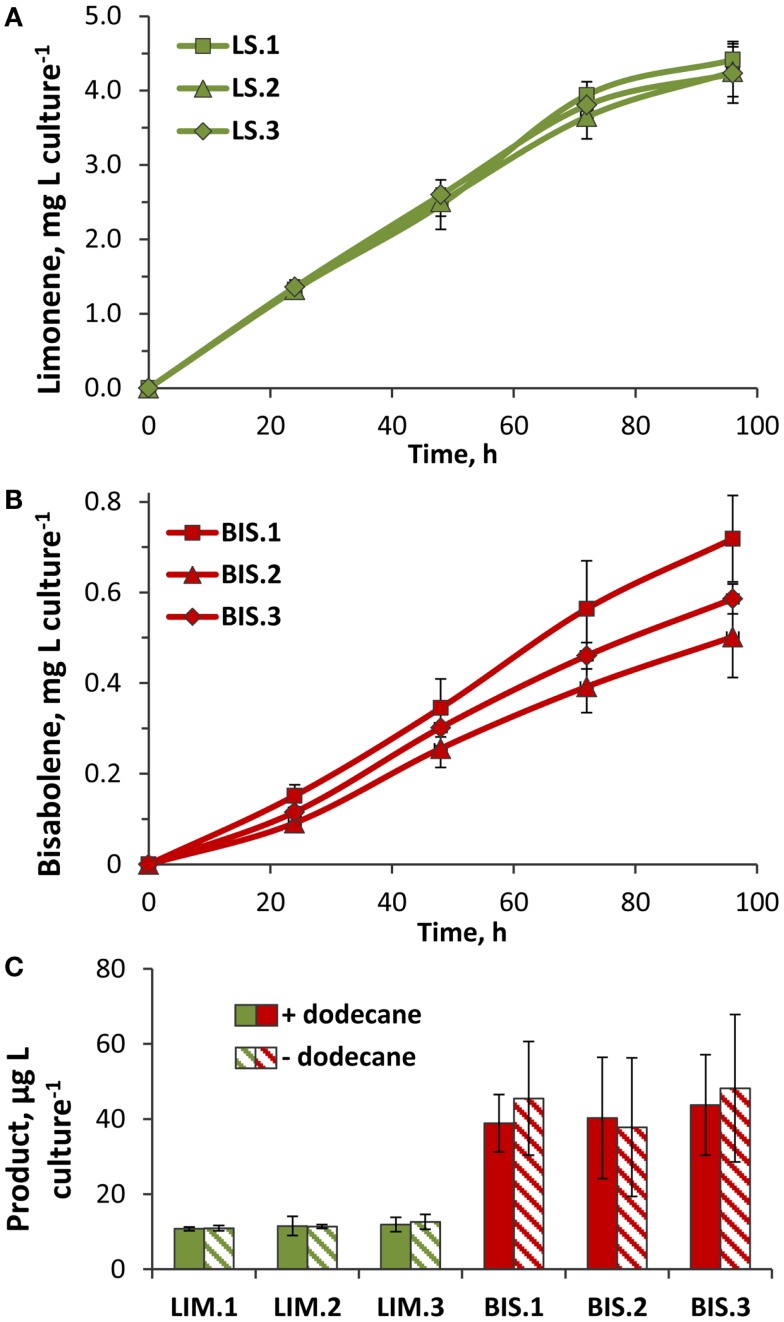
**Rates of limonene and α-bisabolene production in transformant lines**. **(A)** Limonene accumulation in three independent LS transformant lines (LS.1, LS.2, and LS.3). Yields were quantified by GC–FID analysis of limonene extracted by a dodecane overlay during the 96-h growth phase (Figure 5A, +dodecane). **(B)** α-Bisabolene extracted from three independent BIS lines (BIS.1, BIS.2, and BIS.3) over the 96-h growth phase (Figure 5B, +dodecane) using a dodecane overlay. **(C)** Yields of limonene and α-bisabolene extracted from cells harvested after 96 h of photoautotrophic growth (Figure 5) in the presence (solid bars) or absence (hatched bars) of a dodecane overlay. Terpenoids were extracted from cells using a methanol:chloroform extraction method, and quantified by GC–FID. Error bars represent standard deviation from at least three biological replicates.

Three independent lines of the LS transformant consistently produced yields over 4 mg limonene L culture^−1^, with the highest rate of 50 μg L culture^−1^ h^−1^ recorded over the exponential growth phase when cells were actively dividing (Figure 6A). As cultures reached higher cell densities, light limitation due to cell shading began to limit photosynthetic growth (Figure 5A), which likely translated into reduced rates of limonene biosynthesis. Assuming that carbon comprised ~50% of the total biomass, the carbon partitioning to limonene was estimated at 0.3%. Yields of α-bisabolene were considerably lower, where average yields in BIS transformant lines ranged between 0.5 and 0.7 mg α-bisabolene L culture^−1^ (Figure 6B). The highest α-bisabolene production rate was 6 μg L culture^−1^ h^−1^ over the active growth phase, equivalent to ~0.06% of assimilated carbon partitioning as α-bisabolene.

A methanol:chloroform extraction was performed on cells harvested at the termination of the 96-h time course to assess whether any limonene or α-bisabolene remained in the cells after the dodecane extraction procedure. On average, 11 μg limonene L culture^−1^ was extracted from LS cells (representing 0.25% of the total limonene yield), and 40 μg α-bisabolene L culture^−1^ from BIS cells (8% of the total bisabolene yield) (Figure 6C). Therefore, almost 2.5-fold (mol:mol) more α-bisabolene remained inside cells after dodecane extraction, compared to limonene. Relative to the overall terpenoid yields from LS and BIS transformants, a much greater fraction of α-bisabolene was retained intracellularly compared to limonene. This may be explained, in part, by the mass difference between these two compounds, with the smaller limonene molecule having a greater rate of diffusion across the cell membrane.

Surprisingly, similar amounts of limonene and α-bisabolene were extracted from harvested cells regardless of the presence or absence of the dodecane overlay (Figure 6C). Additional dodecane extractions of the spent media performed after cell harvesting did not reveal any terpenoids that partitioned to the media. The higher overall product yields displayed by cultures overlayed with dodecane may be due to: (i) the trapping and solubilization of limonene and α-bisabolene, which prevented evaporative loss, and/or (ii) the sequestration of limonene and α-bisabolene from the cells, which alleviated a negative feedback inhibition mechanism.

### Physiological characterization of *Synechococcus* sp. PCC 7002 Δ*glgC*

**Figure 7:**
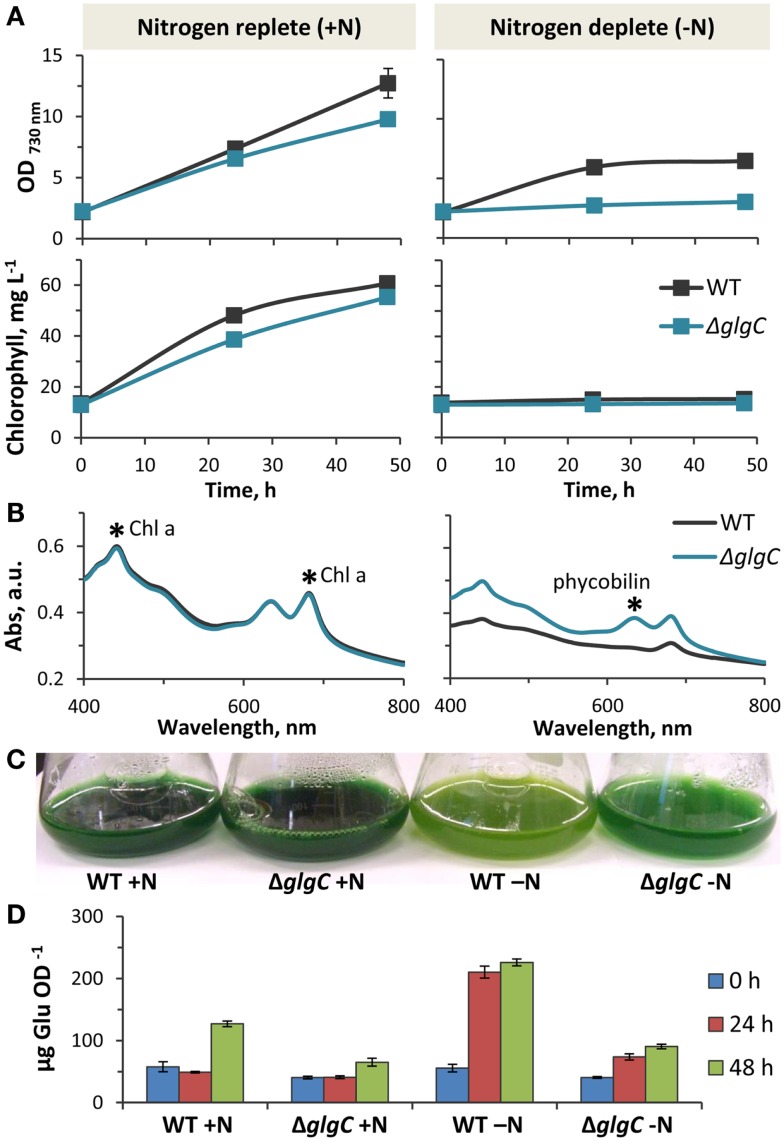
**Physiology of *Synechococcus* sp. PCC 7002 Δ*glg*C**. **(A)**. Comparison of photoautotrophic growth rates in wild type and Δ*glgC* cultures in nitrogen-replete and nitrogen-deplete media, as measured by changes of optical density (730 nm) and chlorophyll content over 48 h. **(B)** Whole cell absorption spectra of wild type and Δ*glgC* cells after 48 h growth in nitrogen-replete and nitrogen-deplete media. **(C)** Differences in pigmentation of wild type and Δ*glgC* cultures after 48 h growth in the presence or absence of nitrogen in the media. **(D)** Carbohydrate content (reported as glucose equivalents) of wild type and Δ*glgC* cells at the different stages of growth (0, 24, and 48 h) shown in **(A)**. Error bars represent standard deviation from three biological replicates, and are hidden beneath the marker if not apparent.

At a light intensity of 250 μmol photons m^−2^ s^−1^ PAR, the Δ*glgC* strain displayed slightly impaired photoautotrophic growth in nutrient-replete media, as measured by OD_730 nm_ and chlorophyll content (Figure 7A, left panels). In contrast to the wild type, Δ*glgC* did not grow photoautotrophically in nitrogen-deplete medium (Figure 7A, right panels) due to its inability to degrade the light-harvesting phycobilisomes as a source of nitrogen (Carrieri et al., 2012; Grundel et al., 2012; Guerra et al., 2013; Hickman et al., 2013). Accordingly, the absorbance spectrum of Δ*glgC* cells in nitrogen-deplete media shows the presence of phycobilin, which is absent in the wild type after nitrogen-deprivation (Figure 7B). As a result, the Δ*glgC* culture retained the blue–green hue under nitrogen-deprivation, while the wild type appeared more yellow–green due to the unmasking of chlorophyll *a* as the blue-pigmented phycobiliproteins were degraded (Figure 7C). We confirmed that the reducing carbohydrate content of Δ*glgC* was diminished compared to the wild type, particularly during stationary phase at 48 h in nutrient-replete media, and also during the first 24 h after the onset of nitrogen-deprivation (Figure 7D). These data confirmed that Δ*glgC* is defective in glycogen biosynthesis, and that our experimental conditions replicated the phenotypes previously observed in Δ*glgC* mutants of other cyanobacterial species (Carrieri et al., 2012; Grundel et al., 2012; Guerra et al., 2013; Hickman et al., 2013), and in *Synechococcus* sp. PCC 7002 (Guerra et al., 2013).

### Organic acid excretion in Δ*glgC* and its effect on terpenoid yields

**Figure 8:**
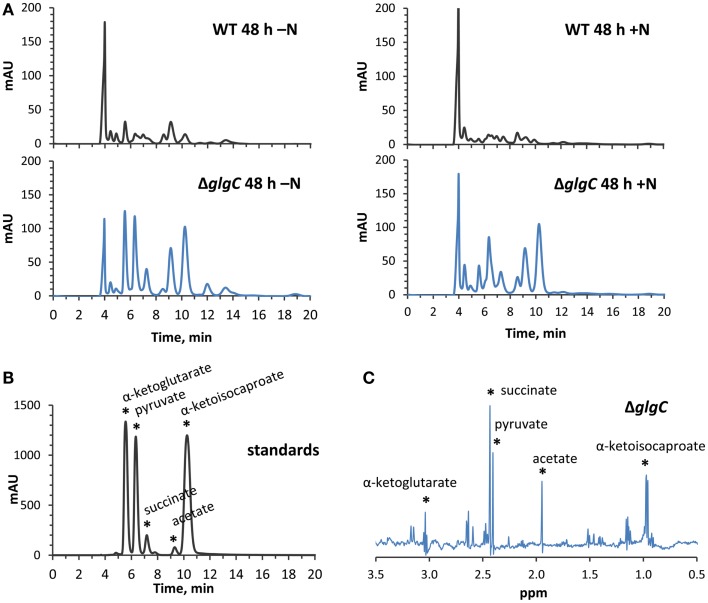
**High-performance liquid chromatography and NMR identification of secreted organic acids from Δ*glgC***. **(A)** HPLC-generated chromatograms showing the presence of secreted metabolites in Δ*glgC*, which are absent from the wild type culture media, when grown for 48 h under nitrogen-deplete (left panels) and nitrogen-replete (right panels) conditions. **(B)** The retention times of the metabolites is shown relative to a mixture of 4 mM α-ketoglutarate, pyruvate, succinate, acetate, and α-ketoisocaproate standards. **(C)** Proton NMR spectrum of the secreted metabolites in Δ*glgC*, showing chemical shifts and peak splitting patterns that correspond to α-ketoglutarate, pyruvate, succinate, acetate, and α-ketoisocaproate.

Consistent with studies in *Synechocystis* sp. PCC 6803 (Carrieri et al., 2012; Grundel et al., 2012) and *Synechococcus elongatus* PCC 7942 (Hickman et al., 2013), we identified pyruvate, α-ketoglutarate, and succinate in the spent media of nitrogen-deprived *Synechococcus* sp. PCC 7002 Δ*glgC* cultures using HPLC analysis (Figure 8A, left panels), through a comparison of elution times with known standards (Figure 8B). However, we also detected α-ketoisocaproate and acetate within the same concentration range, metabolites which were not previously reported in the Δ*glgC* strains of the other cyanobacterial species. Acetate is a common fermentation product putatively derived from acetyl-CoA in cyanobacterial metabolism (Xu et al., 2013), while α-ketoisocaproate is the immediate precursor to leucine. This highlights subtle metabolic distinctions in the metabolism of the Δ*glgC* mutants between the model cyanobacterial species. An additional unique observation was the excretion of the same organic acids by Δ*glgC* in nutrient-replete media at the onset of stationary phase, when wild type cells would normally begin to increase glycogen stores for energy reserves (Figure 8A, right panels). However, on a per-cell basis, these concentrations were much lower relative to the concentrations of those excreted during nitrogen starvation. NMR was used as a second, independent method to verify the identity of these metabolites (Figure 8C). Pyruvate, succinate, and α-ketoisocaproate accumulated at concentrations of ~250 μM, while α-ketoglutarate accumulated at ~200 μM, and acetate ~500 μM under nitrogen starvation.

To assess whether the metabolic flux imbalance in Δ*glgC* redirected metabolites toward terpenoid biosynthesis, we grew the Δ*glgC*:LS and Δ*glgC*:BIS transformants under conditions of nitrogen starvation. The total yields of limonene and α-bisabolene were extremely low relative to the culture volume, which was not surprising given the absence of growth during nitrogen starvation (data not shown). However, terpenoid yield relative to biomass was relatively constant, regardless of whether cultures were grown in the presence or absence of nitrogen. In fact, throughout this study, terpenoid yield as a function of biomass remained relatively constant among all combinations of genetic background (±*glgC*), nutrient availability (±nitrogen), and growth phase (exponential vs. stationary). This indicates that terpenoid biosynthesis is coupled to cell growth and that flux through the terpenoid pathway is tightly regulated, even in the face of metabolic perturbations that increase feedstock availability.

**Figure 9:**
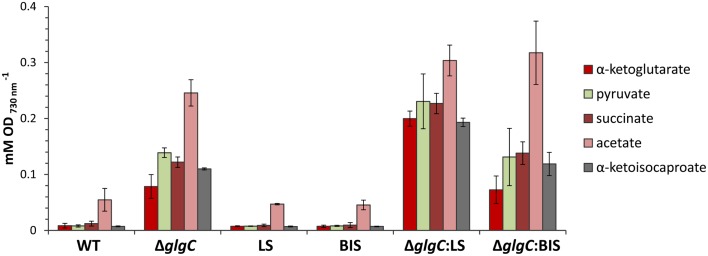
**Accumulation of excreted organic acids in the spend media of cells grown under nitrogen starvation for 48 h**. Concentrations of organic acids were measured by HPLC analysis and presented relative to cell optical density at 730 nm. Error bars represent standard deviation from three biological replicates.

Interestingly, however, we observed a higher extracellular concentration of excreted organic acids in the limonene-producing Δ*glgC*:LS strain, relative to the Δ*glgC* and Δ*glgC*:BIS strains (Figure 9). Under nitrogen starvation, Δ*glgC*:LS accumulated ~2.5-fold more α-ketoglutarate, and almost twofold more α-ketoisocaproate, pyruvate, and succinate in the spent media compared with the Δ*glgC* strain, despite no detectable photoautotrophic growth in either strains. It is unclear whether the higher concentrations in the Δ*glgC*:LS strain are due to increased metabolism toward these end-products, or if a greater proportion of the organic acids are excreted from the cell, but provides an interesting line of future investigation.

## Discussion

We have demonstrated for the first time, the synthesis of limonene in *Synechococcus* sp. PCC 7002, and the first synthesis of α-bisabolene in a photosynthetic microorganism. The choice of *M. spicata* (−)-4*S*-limonene synthase and *A. grandis* (*E*)-α-bisabolene synthase as representative monoterpene and sesquiterpene synthases was based on proven successes in heterotrophic microbial production hosts (Carter et al., 2003; Peralta-Yahya et al., 2011; Alonso-Gutierrez et al., 2013; Ozaydin et al., 2013).

Importantly, (−)-4*S*-limonene synthase and (*E*)-α-bisabolene synthase are known to have high product specificity (Alonso et al., 1992; Rajaonarivony et al., 1992; Bohlmann et al., 1998), which is unusual for TPSs. Most TPSs generate multiple products from the prenyl diphosphate substrate as a result of the highly unstable carbocationic intermediates generated during the electrophilic reaction. Accordingly, these enzymes also produced pure l-limonene and α-bisabolene products in *Synechococcus* sp. PCC 7002; however, the yield of l-limonene was much greater than that of α-bisabolene. This could be explained by the superior enzyme kinetics of the *M. spicata* l-limonene synthase, which has a *K*_m_ (GPP) of 1.8 μM and *k*_cat_ of 0.3 s^−1^ (Alonso et al., 1992; Rajaonarivony et al., 1992), compared to the *A. grandis* (E)-α-bisabolene synthase, with a high *K*_m_ (FPP) of 49.5 μM and very low *k*_cat_ of 0.11 min^−1^ (McAndrew et al., 2011). Substrate availability is also likely to play a significant role because monoterpenes and sesquiterpenes are absent in most cyanobacteria, and so GPP and FPP substrates may not accumulate to the levels seen in plants. However, GPP must accumulate in significant quantities as the precursor to GGPP, from which longer chained cyanobacterial terpenoids are synthesized (e.g., carotenoids), and so may also explain the greater l-limonene yield relative to α-bisabolene.

The cyanobacterium *Synechocystis* sp. PCC 6803 has previously been engineered to produce β-phellandrene monoterpenes (Bentley et al., 2013) and β-caryophyllene sesquiterpenes (Reinsvold et al., 2011). The yield of β-phellandrene was 133 μg gDCW^−1^, as measured by a non-continuous heptane-overlay extraction method. Intracellular titers of β-caryophyllene were ~4 μg gDCW^−1^, as measured by a methanol:choloroform extraction method. However, these yields may have been underestimated due to the volatile nature of the terpenoid products. Evaporation was attributed to major losses of the amorphadiene sesquiterpene from *E. coli* liquid cultures, and a dodecane overlay was demonstrated to reduce the loss (Newman et al., 2006). Recently, the nitrogen-fixing cyanobacterium *Anabaena* sp. PCC 7120 was engineered for limonene production via heterologous expression of the *Picea sitchensis* limonene synthase (Halfmann et al., 2014). The total yield of limonene (114 μg L^−1^) was found to partition as a volatile gas in the culture headspace, and titers were improved ~ninefold upon overexpression of three rate-limiting enzymes involved in terpenoid metabolism. Here, we report the yields of 1.7 mg gDCW^−1^ (4 mg L^−1^) of limonene and 0.3 mg gDCW^−1^ (0.6 mg L^−1^) of α-bisabolene in *Synechococcus* sp. PCC 7002, upon expression of the single TPS genes, and using a continuous dodecane overlay extraction method that may reduce product loss due to evaporation.

It is also conceivable that dodecane may actively sequester terpenoids from the cell, thereby relieving any negative feedback pressures exerted by the products thus promoting higher end-product yields. Indeed, a 20% (v/v) dodecane overlay was found to alleviate growth inhibition of *Synechocystis* sp. PCC 6803 that was induced by the addition of commercial farnesene at concentrations as low as 0.04% (v/v) (Hellier et al., 2013). Further support for this theory was demonstrated in *E. coli*, where heterologous expression of an export pump in a limonene-producing strain increased limonene yield by ~60% (Dunlop et al., 2011). The microbial toxicity of bisabolene appears to be very low, with concentrations of commercial bisabolene up to 20% (v/v) exerting no effect on cell growth in *E. coli* and yeast (Peralta-Yahya et al., 2011). Although toxicity information is useful in this respect, the product feedback inhibition may not always manifest as growth inhibition. To fully understand the increased limonene and α-bisabolene yields in the presence of dodecane, investigations are required: (i) to probe terpenoid (or other) pathway inhibition by these end-products as well as (ii) to quantify the biologically produced limonene and bisabolene lost via evaporation in the absence of dodecane. It is important to establish whether the product naturally separates from biomass and how the physical properties of the product (mass, boiling point) influence this, or whether the product exerts feedback inhibition so that engineering efforts may be targeted toward this.

**Figure 10:**
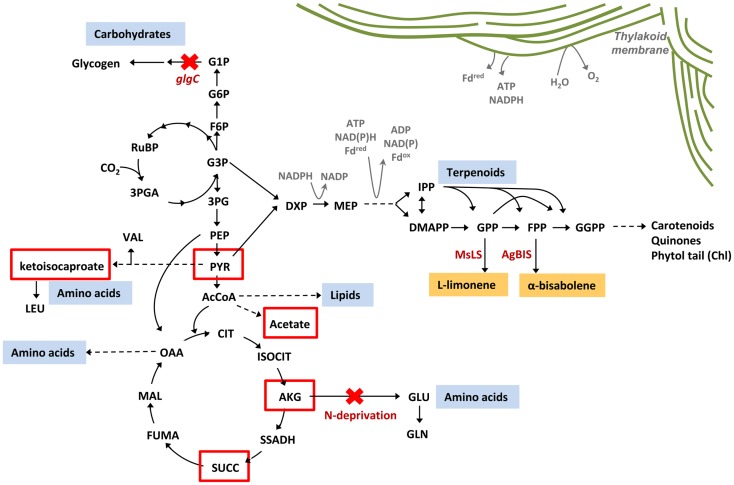
**Metabolic redistribution in *Synechococcus* sp. PCC 7002 upon simultaneous blocks to protein and carbohydrate sinks**. Protein (amino acids), carbohydrates (mainly glycogen), and lipids represent the major metabolic sinks in *Synechococcus* sp. PCC 7002. When metabolic flux to glycogen and amino acids were simultaneously blocked, through inactivation of Δ*glgC* and elimination of nitrogen from the culture medium (red crosses), a suite of organic acids were secreted from the cell as overflow metabolites from central metabolism (red boxes). There is potential to redirect these spill-over intermediates toward biotechnologically relevant pathways to increase yields of engineered products, such as limonene and α-bisabolene via the terpenoid biosynthetic pathway.

Targeted metabolic flux redistribution among the major carbon sinks is the key to producing novel end-products from engineered microbes at commercially available quantities. The goal remains to increase product yield at the expense of biomass accumulation (cell growth and division), without negatively impacting metabolisms associated with the assimilation and direction of carbon to the product. Although disruption of glycogen biosynthesis in *Synechococcus* sp. PCC 7002 produced an oversupply of central metabolites (α-ketoisocaproate, α-ketoglutarate, pyruvate, succinate, and acetate) (Figure 10), there was no apparent increase in flux though the MEP terpenoid pathway as conveyed by yields of limonene and α-bisabolene reporter products. Furthermore, such a severe perturbation of central carbon metabolism had a detrimental effect on the organism fitness as manifested by a complete growth inhibition. To that end, introduction of alternative routes for carbon partitioning have proven successful in cyanobacteria as carbon fixation rates are stimulated by providing alternative sinks for photosynthetically derived NADPH and ATP (Ducat et al., 2012; Li et al., 2014). While the MEP terpenoid pathway utilizes both NADPH and ATP as cofactors, growth and pathway optimization are required so that photosynthesis and terpenoid biosynthesis remain active despite major metabolic redistributions.

## Conflict of Interest Statement

The authors declare that the research was conducted in the absence of any commercial or financial relationships that could be construed as a potential conflict of interest.
